# Designing effective U = U communication strategies considering the needs of PLHIV, their partners, and healthcare worker constraints in South African clinics

**DOI:** 10.1371/journal.pone.0295920

**Published:** 2023-12-20

**Authors:** Dorina Onoya, Tembeka Sineke, Rachel King, Idah Mokhele, Shubhi Sharma, Mandisa Dukashe, Refiloe Cele, Dorah Bokaba, Robert Inglis, Simangele Sigasa, Jacob Bor

**Affiliations:** 1 Health Economics and Epidemiology Research Office, Faculty of Health Sciences, University of the Witwatersrand, Johannesburg, South Africa; 2 Faculty of Psychology and Neuroscience, Maastricht University, Maastricht, The Netherlands; 3 Institute for Global Health Sciences, University of California, San Francisco, San Francisco, CA, United States of America; 4 Department of Global Health, Boston University School of Public Health, Boston, MA, United States of America; 5 South African National AIDS Council (SANAC), Pretoria, South Africa; 6 Tshwane Department of Health, Tshwane, South Africa; 7 Jive Media Africa, Pietermaritzburg, South Africa; Human Sciences Research Council, SOUTH AFRICA

## Abstract

**Introduction:**

We sought to understand the Undetectable = Untransmittable (U = U) communication needs of persons living with HIV (PLHIV) and barriers to U = U communication among healthcare providers (HCPs) in South Africa.

**Methods:**

We conducted five focus group discussions (FGDs) with HCPs (N = 42) including nurses and counsellors from primary healthcare clinics (PHCs) in the Gauteng and Free State Provinces of South Africa, three FGDs (N = 27) with PLHIV recruited by snowball sampling from civil society organizations, and 27 in-depth interviews (IDIs) with recently diagnosed PLHIV in Johannesburg. IDIs and FGDs were audio recorded, transcribed, translated to English, and analysed thematically.

**Results:**

PLHIV were largely unaware and sceptical of U = U as the message appeared to contradict the mainstream HIV prevention clinical guidance. The low viral load (VL) knowledge further reduced confidence in U = U. PLHIV need support and guidance on the best approaches for sharing U = U information and disclosing their VL status to their partners, highlighting the central role of community understanding of U = U and VL to mediate the desired stigma reduction, social acceptance and emotional benefits of U = U for PLHIV. HCPs were uneasy about sharing U = U due to concerns about risk compensation and ART non-adherence and worried about enabling any ensuing HIV transmission. HCPs also need a simple, unambiguous, and consistent narrative for U = U, integrated with other HIV prevention messages. PLHIV and HCPs alike recommended a patient-centred approach to communicating U = U, focusing primarily on attaining viral suppression and emphasizing that condomless sex is only safe during periods of ART adherence.

**Conclusions:**

These data highlight the need for simple U = U communication support targeting both HCP and PLHIV. Culturally appropriate communication materials, with training and ongoing mentorship of the clinic staff, are essential to improve patient-centred U = U communication in clinics.

## Introduction

Antiretroviral therapy (ART) eliminates the risk of HIV transmission from a virally suppressed person receiving treatment to their sexual partner [1–4]. Although South Africa moved to immediate ART for all persons diagnosed with HIV6, treatment uptake and viral load (VL) testing remain suboptimal [5, 6]. Challenges in ART adherence, retention, and HIV stigma contribute to the disconnect between individual-level efficacy and population-level effectiveness of “treatment-as-prevention” (TasP) [7–9]. However, the benefits of TasP are not systematically conveyed to patients as part of HIV counselling in South Africa, limiting the potential of this information to motivate treatment adherence, improve viral suppression, and reduce stigma [10, 11]. The poor impact of TasP on individual HIV treatment outcomes may be partially attributable to the low and indirect dissemination of the prevention benefits of ART [12, 13].

In 2016, the Prevention Access Campaign launched the Undetectable = Untransmittable (U = U) campaign to promote the dissemination of TasP, focusing on the social, mental, and emotional benefits of eliminating HIV transmission fears by emphasising viral suppression. The direct communication of U = U has been shown to increase men’s HIV testing uptake in South Africa [14], improve persons living with HIV (PLHIV)’s self-image, and reduced anticipated HIV social stigma related to dating and sex [15, 16]. However, knowledge about the U = U campaign and the science of TasP more generally is low in Sub-Saharan Africa where the HIV burden is highest [10], with countries’ governments hesitant to endorse the U = U message for dissemination within healthcare settings [11]. Yet, U = U communication by a healthcare provider (HCP) was shown to have a superior impact on adherence, higher self-reported viral suppression, and HIV disclosure than U = U dissemination in non-healthcare settings [17].

Given the potential for U = U communication to improve the psychological well-being of PLHIV and boost progress toward HIV treatment and prevention goals, it is essential to ensure that the U = U is communicated widely and appropriately in healthcare settings. In this study, we sought to understand the U = U communication needs for PLHIV, as well as barriers and facilitators to U = U communication among HCPs, to inform a communication strategy aiming to improve overall viral suppression and improve well-being among PLHIV in South Africa [13].

## Methods

### Study design and sample

The study was the formative research component of a randomized trial [10, 11] to integrate U = U into HIV counselling in South Africa (ClinicalTrials.gov ID: NCT04504357; NIH grant #: R34MH122323). The study consisted of in-depth interviews and focus group discussions with PLHIV, HIV counsellors, and clinic nurses.

### Data collection

For each of the three participant type, we used convenient sample of participants

PLHIV participants (Table 1): We conducted three focus group discussions (FGD) with 27 ART-experienced PLHIV recruited through snowball sampling via peer-support networks, including six from a U = U advocacy group, in Johannesburg, South Africa. In addition, we conducted in-depth interviews with a separate group of 27 newly diagnosed adult (≥18 years) PLHIV, recruited by referral from the attending lay counsellor, after their post-HIV test counselling at three public primary healthcare clinics (PHC) in Johannesburg. In-depth interviewing (IDI) was the preferred method for this group due to privacy considerations for newly diagnosed PLHIV. All FGDs were facilitated by two trained staff and lasted approximately two hours. The IDIs lasted approximately 60 minutes and covered similar topics as the FGDs. The interview guides for the FGD and IDI with PLHIV gauged PLHIV’s knowledge about U = U, VL suppression and U = U, motivation for communicating/using HIV treatment for prevention, sexual relationships, and questions about provider communication on U = U.

**Table 1:** Overview of PLHIV participants.

	IDIs (post-HIV diagnosis)	FGD1 (Peer support network)	FGD2 (U = U advocates)	FGD3 (Peer support network)
	N = 27 (col%)	N = 9 (col%)	N = 6 (col%)	N = 12 (col%)
**Age at enrolment**				
18–30	24 (88.9)	0	6 (100.0)	0
30+	3 (11.1)	9 (100.0)	0	12(100.0)
**Gender**				
Females	23 (85.1)	9 (100.0)	6 (100.0)	N/A
Males	4 (14.9)	N/A	N/A	12(100.0)
**Recruitment source**				
Civil society organisation	0	9 (100.0)	6 (100.0)	12(100.0)
Primary health facility	27 (100.0)	0	0	0

Public-sector healthcare providers (HCP): We conducted two focus group discussions (FGD) among lay HIV counsellors (n = 10) and among nurses (n = 12) from eight PHCs in Johannesburg (Table 2). All healthcare worker participated voluntarily after referral by their respective facility managers. The FGDs lasted approximately 60 minutes. The FGD guide for HCP enquired about their U = U knowledge, attitude, processes for communicating VL results and U = U to new and returning PLHIV, and barriers and opportunities for U = U communication in primary healthcare.

**Table 2:** Overview of HCP participants.

Type of provider	Total
Lay counsellor trainers/supervisors	28
PHC nurses	12
Project Coordinator	3
Social auxiliary workers	6
Enrolled nursing assistant	1
Technical advisor: Psychosocial support	2
Peer educators	2
Community Systems Technical Officer (CSTO)	10
**Total**	**64**

District support partners (DSP) counsellors and managers: We also conducted four FGDs with PHC managers and counsellors of two major non-governmental organisations (NGOs) supporting the HIV treatment program in the Gauteng and the Free State provinces in South Africa (N = 42). We requested permission to engage five major DSP operating across South Africa and conducted FGD among the staff of the two NGOs that granted permission.

All data were collected between April 2021 and May 2022. All participants provided written consent to participate in the study. All interviews and FGDs were conducted in English, Zulu or Sotho and audio recorded. All audio recordings were transcribed verbatim and translated as necessary before analysis.

### Data analysis

All identifiers were removed from the final analytic data to maintain participants’ confidentiality. All participants provided written informed consent. The anonymised transcripts were analysed thematically. Each transcript was coded by two coders independently, with initial themes drawn from IDI and FGD guides. Major and cross-cutting themes were identified and then refined over several workshops. In cases where differences in codes could not be reconciled, a third member of the research team was assigned to help resolve the differences. This study was approved by the Human Research Ethics Committee (Medical) of the University of the Witwatersrand (M200529 MED20-05-019) and the Boston University Medical Campus Institutional Review Board (H-40891).

## Results

### PLHIV’s Views on U = U Communication

#### PLHIV are mostly unaware and are sceptical about U = U

Newly diagnosed PLHIV understood the health benefits of ART but knew little about the U = U campaign. More knowledgeable, ART-experienced PLHIV were largely sceptical about the safety of condomless sex under U = U. Some participants learned the term U = U during the focus group discussions and were surprised by the potential prevention benefits of ART adherence, particularly the possibility of having condomless sexual relations without the risk of HIV transmission.

“*So*, *when you are virally suppressed*, *I am just trying to understand this*, *because I don’t know this information*. *If you can help me*. *Because I know Prep*… *if you are negative*. *Are you saying when you are virally suppressed; is that what the term is*? *Is it undetectable if it is suppressed*? *“*(Female PLHIV)

For PLHIV, viral suppression conveyed hope, primarily for sustained health and the prevention of HIV transmission to infants. However, the understanding and belief that U = U could normalise sexual relations and protect against transmission were less apparent.

“*I feel happy because I am adhering to my treatment which means I will live a longer life*. *As I am a student right now*, *I lost hope when I found out that I’m HIV positive but as I kept coming to the clinic and the nurses telling me that the treatment is working my viral load is dropping and my baby was fine*, *I started gaining hope”* (Female PLHIV)

#### PLHIV need greater access to VL testing and proactive VL counselling at clinics

Effective U = U messaging presupposes baseline treatment literacy including an accurate understanding of VL suppression and its relationship to HIV transmission. However, PLHIV expressed that VL testing was not always emphasized and that scientific information about VL suppression and its implications was not always shared during counselling with greater emphasis on the impact of ART on the CD4 count. PLHIV may be told that they are “virally suppressed” without explanations about the meaning of this status and its implications for sexual transmission.

“*Because I see it as a challenge because we say U = U*, *yes*, *we know that it works*. *We know that it’s undetectable*, *if I make sure that I take my treatment and adhere*, *I am not going to transmit HIV*. *But when you go to the facility*, *they don’t give you the VL test*. *They don’t test you and even if they do test you*, *they’re not going to communicate or translate the results or interpret them to make sure that you understand*.*”* (Female PLHIV)

Among PLHIV who understood U = U, a VL-suppressed result was a measure of ART’s effectiveness and a reward for adherent behaviour. Achieving and maintaining VL suppression was therefore an important motivator and target to return to a “normal human” status, particularly concerning sexual partnerships and childbearing, eliminating the fear of HIV transmission. Although PLHIV freely acknowledged temporary lapses in adherence, they were uncertain about the exact impact of brief treatment interruptions on their VL suppression status, especially in the absence of regular testing. PLHIV were less familiar with the VL testing schedule and did not plan for it, as these tests were mostly HCPs initiated.

#### PLHIV are HIGHLY motivated to avoid HIV transmission, but inconsistent condom use persists

PLHIV were very motivated to prevent onward HIV transmission but were more confident about the role of condom use in the prevention of sexual HIV transmission. Male PLHIV participants were more candid about reducing the frequency or even forgoing condom use as relationships progressed. PLHIV who believed U = U much preferred ART over condoms as the primary prevention strategy, especially in long-term relationships.

“*But I once had a relationship where I spent two years*, *two full years*, *we did not skip the condom*, *not even once*, *because she knew my status*. *But it was only in this one relationship*. *The rest of my relationships*, *after three days*, *four days*, *nah…*.*”* (Male PLHIV)

“*No*, *no*, *no*. *I rather skip condom than my pills*. *Because if the viral load is suppressed*, *very slim chances that I can infect someone*.*”* (Male PLHIV)

Inflexible and persistent condom messaging, even for PLHIV who are stable on ART and virally suppressed, has sustained the burdensome belief that PLHIV would always be infectious and condom use must be maintained. While community organisations sometimes provide information on U = U, these messages are not consistently reinforced by governmental clinic service providers. This inconsistent messaging by different HCPs casts doubts on the science, contributing to U = U scepticism.

#### HIV status disclosure support is needed to maximise the benefits of U = U

Although HIV disclosure is challenging, PLHIV felt that U = U could make it easier. Telling a potential partner that they were “HIV virally suppressed” instead of simply “HIV-positive” signalled that they were “responsible” and “not careless” with their health and that of their partners. Nevertheless, most participants maintained that disclosure was difficult when the broader community lacked an understanding of VL suppression and U = U. Yet, educating their partner about U = U at the same time as the disclosure was perceived as a significant burden, and consistent communication of U = U through digital and mainstream media are essential to mitigate community and partners’ fears of HIV transmission.

“*U = U gave me hope*, *it gave me hope to live*. *Not just to live*, *but to live a healthy and productive life*. *I’m still a bit sceptical when it comes to informing people*, *and sharing information about the benefits of taking medication to a point that you can suppress viral load and not be able to infect the next person*. *I think it should be given to people based on their level of understanding”* (Male PLHIV)

### HCP’s Perspectives on U = U Communication

#### U = U was perceived to contradict guidance on condom promotion

HCP knowledge of the prevention benefits of ART was uneven, with nurses more aware of U = U and lay counsellors being more uncertain. Lay counsellors were particularly uneasy and preferred to defer questions on transient treatment interruptions and resulting viremia to nursing staff. HCP were nearly unanimous in emphasising the importance of standardised U = U communication training for any persons tasked with encouraging HIV treatment adherence.

“*How do you communicate saying*, *well if you take your ARVs correctly then you can stop using a condom*? *How do you communicate back without worrying that it might be misconstrued by the client and thinking okay let me just stop using a condom*?*”* (Nurse)

HCPs reiterated the need to balance U = U with condom use promotion and were very reluctant to share information that seemed contradictory to the approved strategy for the prevention of sexually transmitted infections (STI).

“*You are virologically suppressed*, *this other person*, *his/her viral load is unknown or it’s high*. *The chances of you getting reinfected (with a new*, *potentially resistant strain of HIV) and for your viral loads to go high*, *are still there*. *So*, *hence we are saying we promote condom usage*.*”* (Nurse)

#### Healthcare workers fear being unwittingly responsible for HIV transmission events

HCPs were also uncertain about patients’ ability to understand U = U information and to apply it safely in their daily lives. They doubted that most PLHIV could be trusted to manage the knowledge that viral suppression can also mean freedom from barrier sexual protection methods. Mostly, HCPs feared being indirectly responsible for HIV transmissions resulting from risk compensation behaviours or even incorrect assessment of viral suppression in the absence of regular viral load testing.

*“I don’t think I’ll be comfortable telling the patient about U = U because we know the behaviour of patients who have that information*. *Then it means we’ll be telling them to go*, *you know*, *having unprotected sex with all the unknown partners*.*”* (Lay counsellor)

#### Need for clear guidance for patient-centred and goal-oriented viral load counselling

Both HCP and PLHIV favoured communicating U = U, including the possibility of condom-free sex, in a patient-centred approach, tailoring communication to individual patients’ circumstances and historical adherence behaviour. Both PLHIV and HCP favoured clear U = U communication emphasising the benefits of ART for the PLHIV first, before adding benefits for others. Secondly, participants recommended strengthening PLHIV’s understanding and demand for viral load tests and viral suppression as a measure of adherence (VL literacy). Thirdly, messaging on the expanded VL suppression prevention benefits, including the possibility of condom-free sex as a reward for sustained adherence, should be determined in a patient-centred approach.

“*When we see that there is progress*, *then we are going to encourage this particular person to keep it up for VL to go lower*. *We are looking at their benefits which is their health at that time*. *We are not talking about the external benefits which would be not infecting other people*. *So right now*, *you are healthier*, *like when you have flu*, *you should not worry that much because it is getting better*. *And then for it to get even better*, *continue with your treatment*. *Once they are healthy*, *that is when you can talk about whether a person can look up to some point in time when they cannot use condoms and just enjoy sex with their partners*.*”* (PLHIV counsellor)

## Discussion

Despite the clear science of U = U, formal endorsement by national governments and its incorporation in HIV prevention messaging in sub-Saharan Africa have been slow. To our knowledge, this is one of the first studies to provide an in-depth exploration of the meaning of U = U for PLHIV and HCPs, and the barriers and facilitators to expanding U = U communication in South Africa.

Similar to previous studies, we found that PLHIV understand the health benefits of ART, with significant gaps in knowledge and only moderate confidence in U = U [10, 18, 19]. The scepticism was rooted in PLHIV’s limited VL literacy and challenges in understanding the implications of U = U for their relationships [20, 21]. While the U = U approach to VL communication can facilitate HCPs’ partnerships with patients to improve their self-management, the potential benefits of U = U on ART adherence and the mental and emotional well-being of PLHIV are likely mediated by U = U belief among current and prospective partners [21, 22]. However, HIV-negative/naïve persons in South Africa are largely unaware or sceptical of U = U [18]. A recent study reported that HIV-negative partners were unwilling to stop PrEP or condom use even after their partners achieved viral suppression [23]. The potential benefits of U = U on ART adherence behaviours and PLHIV wellbeing are likely mediated by U = U knowledge among current and prospective partners [21]. Indeed, this was confirmed in a randomized trial in Malawi: disseminating information on TasP at the community level increased HIV testing by increasing people’s beliefs about *other community members’* TasP knowledge and reducing anticipated stigma. It is therefore necessary for U = U campaigns to combine clinical counselling with wider community-based dissemination to increase the VL literacy of communities and persons in PLHIVs’ network.

The hesitancy of HCP to disseminate U = U information is underscored by the lack of a concise and approved narrative for U = U counselling and fear of reprisal [24]. The lack of a government-approved U = U communication strategy also leads to inconsistent messaging across the landscape of government and private/NGO-supported HCPs [25, 26]. HCPs are also concerned about PLHIV’s behavioural response to U = U, given current challenges with treatment adherence [27]. Healthcare workers need support to adequately communicate U = U, adapting the message to individual patients’ lived experiences and health-seeking behaviours while remaining scientifically accurate and mitigating the potential for misunderstanding. However, there is no formal process or tool to assess and manage PLHIV’s readiness for U = U information or narrative integrating U = U with established STI and pregnancy prevention messaging for PLHIV, their partners and the broader communities in South Africa.

### Limitations

While the information from HCPs was more broad-based, interviews with PLHIV were limited to the City of Johannesburg, and perspectives may differ in other areas of the country. Further research extending beyond the Gauteng province and even outside South Africa is essential to yield more universally applicable results, as communication strategies may require context-specific adaptations. Also, ART-experienced PLHIV recruited via U = U advocacy organizations likely has better-than-average awareness of U = U. Finally, as we informed participants that we were interested in designing an intervention to disseminate information on U = U, there was potential that desirability bias could shape responses by making people more favourable to U = U dissemination. In this light, the barriers that were identified are that much more significant to address.

## Conclusions

With growing calls for U = U dissemination in South Africa, it is necessary to craft locally appropriate messages, modelling how to integrate U = U into people’s lives. Also, for U = U to fully impact the ART cascade targets, it is essential to ensure acceptability among both HCPs and PLHIV by presenting U = U as symbiotic–and not contradictory—to current prevention strategies to achieve the desired population sexual health outcomes. Additionally, to address the concerns of HCP, U = U messaging would need to be integration in policy/ guidelines.

## Supporting information

S1 File (DOCX)
